# Siblings with MAN1B1-CDG Showing Novel Biochemical Profiles

**DOI:** 10.3390/cells10113117

**Published:** 2021-11-10

**Authors:** Nobuhiko Okamoto, Tatsuyuki Ohto, Takashi Enokizono, Yoshinao Wada, Tomohiro Kohmoto, Issei Imoto, Yoshimi Haga, Junichi Seino, Tadashi Suzuki

**Affiliations:** 1 Department of Medical Genetics, Osaka Women’s and Children’s Hospital, Izumi 594-1101, Japan; 2 Department of Molecular Medicine, Research Institute, Osaka Women’s and Children’s Hospital, Izumi 594-1101, Japan; waday@wch.opho.jp; 3 Department of Pediatrics, Tsukuba University Faculty of Medicine, Tsukuba 305-8576, Japan; tohto@md.tsukuba.ac.jp (T.O.); enokizono.takashi.mg@alumni.tsukuba.ac.jp (T.E.); 4 Division of Molecular Genetics, Aichi Cancer Center Research Institute, Nagoya 464-8681, Japan; tomo069medicalgenetic@gmail.com (T.K.); issehgen@gmail.com (I.I.); 5 Department of Human Genetics, Graduate School of Biomedical Science, Tokushima University Graduate School, Tokushima 770-8503, Japan; 6 Glycometabolic Biochemistry Laboratory, RIKEN Cluster for Pioneering Research (CPR), 2-1 Hirosawa, Wako 351-0198, Japan; yoshimi.haga@jfcr.or.jp (Y.H.); jseino@riken.jp (J.S.); tsuzuki_gm@riken.jp (T.S.); 7 Cancer Proteomics Group, Cancer Precision Medicine Center, Japanese Foundation for Cancer Research, 3-8-31 Ariake, Koto-ku, Tokyo 135-8550, Japan

**Keywords:** MAN1B1, intellectual disability, congenital disorders of glycosylation, early-onset epileptic encephalopathy, mass spectrometry

## Abstract

Congenital disorders of glycosylation (CDG), inherited metabolic diseases caused by defects in glycosylation, are characterized by a high frequency of intellectual disability (ID) and various clinical manifestations. Two siblings with ID, dysmorphic features, and epilepsy were examined using mass spectrometry of serum transferrin, which revealed a CDG type 2 pattern. Whole-exome sequencing showed that both patients were homozygous for a novel pathogenic variant of *MAN1B1* (NM_016219.4:c.1837del) inherited from their healthy parents. We conducted a HPLC analysis of sialylated N-linked glycans released from total plasma proteins and characterized the α1,2-mannosidase I activity of the lymphocyte microsome fraction. The accumulation of monosialoglycans was observed in MAN1B1-deficient patients, indicating N-glycan-processing defects. The enzymatic activity of MAN1B1 was compromised in patient-derived lymphocytes. The present patients exhibited unique manifestations including early-onset epileptic encephalopathy and cerebral infarction. They also showed coagulation abnormalities and hypertransaminasemia. Neither sibling had truncal obesity, which is one of the characteristic features of MAN1B1-CDG.

## 1. Introduction

Glycoproteins play important roles in many biological processes, such as growth, differentiation, organ development, signal transduction, and immunological defenses. Congenital disorders of glycosylation (CDG) are inherited metabolic diseases caused by defects in glycosylation. The number of reported CDG cases has rapidly increased to at least 130 [1,2]. Some types of CDG may be diagnosed by the glycosylation pattern of serum transferrin. The biosynthesis of N-linked glycans is a complex process. Two types of CDG related to N-glycan biosynthesis have been identified. Type I defects are caused by abnormalities in the biosynthesis, assembly, and transfer of ER-localized dolichol-linked oligosaccharide Glc_3_Man_9_GlcNAc_2_ to protein. Type II defects are attributed to abnormalities in the processing reactions of protein-bound oligosaccharides. Type II defects include abnormalities in glycosidases, glycosyltransferases, nucleotide sugar transporters, regulators of pH homeostasis, and the trafficking of resident Golgi proteins.

Patients with CDG show various abnormalities that manifest in early childhood. A clinical diagnosis of CDG is often difficult because many organs are affected and there is a lack of clinical uniformity. Neurological manifestations include developmental delays, intellectual disability (ID), cerebellar atrophy, myopathies, strokes and stroke-like episodes (SLE), epileptic seizures (encephalopathy), and demyelinating neuropathy. The clinical manifestations and severity of the disease are often heterogeneous.

Pathogenic variants of *MAN1B1*, which encodes the Golgi mannosyl oligosaccharide α1,2-mannosidase, have been reported [3,4,5,6,7,8,9,10,11,12,13,14,15,16]. MAN1B1-CDG is also called Rafiq syndrome (MIM#614202). MAN1B1-CDG is characterized as a type II CDG, disrupting not only protein N-glycosylation, but also the general Golgi morphology. MAN1B1 contributes to the timing and disposal of misfolded glycoproteins through the endoplasmic reticulum-associated degradation (ERAD) pathway [17]. MAN1B1-CDG is inherited in an autosomal recessive manner and is characterized by ID, dysmorphic features, and truncal obesity. Truncal obesity is unique to this CDG [5,6]. Its impairment results mostly in a relatively mild syndrome. MAN1B1-CDG does not show systemic manifestations, in contrast to PMM2-CDG, and difficulties are associated with its diagnosis without whole-exome sequencing (WES) or an investigation of glycosylation defects using specific methods.

We identified two siblings with MAN1B1-CDG with early-onset epileptic encephalopathy and neonatal cerebral infarction, respectively, which are rare complications in this CDG. They also showed coagulation abnormalities and hypertransaminasemia. We detected the accumulation of monosialoglycans in plasma, indicating N-glycan-processing defects. We also demonstrated that the activity of MAN1B1 was compromised in patient-derived lymphocytes.

## 2. Materials and Methods

### 2.1. Ethical Approval and Informed Consent

Patient data were collected as part of standard clinical care. Informed consent was provided by the participants according to the regulations of the participating institutions in accordance with the Declaration of Helsinki, and the study was approved by the Ethics Committee of each institution.

### 2.2. Clinical Report

**Figure 1:**
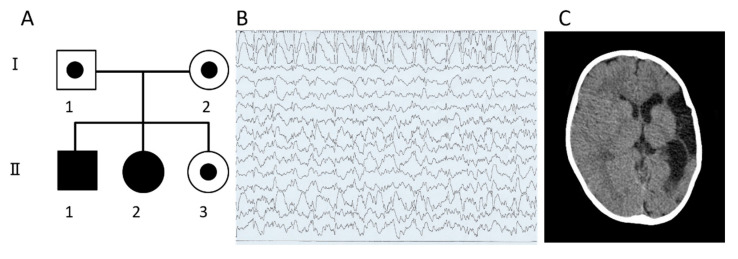
(**A**) Pedigree of the family, (**B**) EEG of patient II-1 at 4 years of age continued to show spikes and waves mainly over the frontal area, (**C**) Brain CT of patient II-2 at 3 years of age showed a massive low-density area in the left cerebral hemisphere, indicating infarction of the left middle cerebral artery.

Patient II-1: The patient (Figure 1A), a 15-year-old boy, was the first child of healthy non-consanguineous Japanese parents. He was born through normal delivery, without asphyxia, and with a birth weight of 3090 g at 39 weeks of gestation. Tachypnea and muscle hypotonia developed at the age of six months, which required admission to the hospital. A physical examination showed delayed motor development, with incomplete head control and rolling due to hypotonia. Two months later, tonic spasms developed, together with hypsarrhythmia on EEG, suggestive of West syndrome. Brain MRI was normal. Treatment with vitamin B6 and valproate resulted in the disappearance of seizures and abnormal EEG findings. However, the exacerbation of West syndrome at the age of 13 months required treatment with ACTH. Routine laboratory tests revealed mildly elevated transaminases. AST ranged from 77–170 IU/L (normal, 8–38 IU/L) and ALT from 58–206 IU/L (normal, 4–44IU/L), while serum direct bilirubin and PT-INR values were within normal limits during infancy. A transient elevation (up to 16,480 IU/L) in the serum level of alkaline phosphatase (ALP) was noted during ACTH therapy; however, various tests did not identify the cause. Although ACTH therapy was mostly successful, focal spikes were persistently observed on EEG, requiring the continuous use of anticonvulsants.

At the age of 15 months, viral infection was associated with increases in transaminase activities and decreases in coagulation activities, such as the prolongation of the prothrombin time (PT)/activated partial prothrombin time (APTT) and low levels of antithrombin (AT), with the following peaks: AST 465 IU/L, ALT 122 IU/L, PT-INR 1.48, APTT 48.4 sec (reference 33.0 sec), and AT activity 29%. Protein C and protein S activities were also 50% lower than normal values. These findings gradually improved within two weeks without symptoms of severe acute liver failure. Horizontal nystagmus appeared at approximately the same time; however, the patient was able to sit unaided, smile at caregivers, and was interested in playing with toys.

He developed slowly thereafter and started to walk unaided at 3 years and speak several words by 4 years. Although EEG continued to show spikes and waves mainly over the frontal area (Figure 1B), he remained free of seizures.

In the latest clinical examination at the age of 15 years, he was unable to compose complex sentences and the intelligence quotient was less than 35. A mild elevation in AST (41–53) was detected, whereas ALT and PT-INR remained within normal limits. Mild dysmorphic features were evident, including hypertelorism, epicanthal folds, down-slanting palpebral fissures, low-set ears, prognathism, thick lips, and microretrognathism. Furthermore, joint hypermobility and scoliosis were noted without truncal obesity or overeating.

Patient II-2: The patient (Figure 1A), an 11-year-old girl and the younger sister of patient II-1, was born at 38 weeks of gestation by Caesarean section free of asphyxia, weighing 2776 g. Mild muscle hypotonia, recurrent apneic episodes, and irritability were noted on the first day of life. Brain CT showed a massive low-density area in the left cerebral hemisphere, indicating infarction of the left middle cerebral artery (MCA) (Figure 1C). Laboratory tests showed normal PT and APTT, with decreased protein C and protein S activities to 50% of normal levels, similar to patient II-1. AST was mildly elevated (up to 66 IU/L), while ALT and direct bilirubin were within normal limits. Further blood tests showed moderately elevated levels of ALP (up to 2556 IU/L). The patient started to walk at the age of 22 months and speak some meaningful words. Protein C and protein S activities increased to the lowest normal limits by 3 years. In the latest visit to the clinic at the age of 11 years, she showed mild ID and a clinical examination revealed various dysmorphic features, similar to those observed in Patient II-1. AST, ALT, and PT-INR values were within normal limits; however, AT activity was still decreased to 73%. No truncal obesity was present. Her sister, II-3, has developed normally so far.

### 2.3. Mass Spectrometry

#### 2.3.1. Purification of Transferrin and Isolation of Glycosylated Peptides

Transferrin was purified from serum by affinity chromatography using rabbit immunoglobulin raised against human transferrin (Dako, Denmark) conjugated to a Hi-Trap NHS-activated agarose column (GE Healthcare, UK). Transferrin bound to the column was eluted with 0.1 M glycine-HCl, pH 2.5. In the analysis of glycopeptides, transferrin was S-carbamidomethylated and digested with 1% (*w*/*w*) each of lysyl endopeptidase (Wako Pure Chemicals, Tokyo, Japan) and trypsin (Promega, Madison, WI, USA) at 37 °C for 16 h. Glycosylated peptides in the digest were enriched using the hydrophilic affinity method according to a previously described method [18]. Briefly, a 10-µg digest was mixed with 15 µL of packed-volume Sepharose CL-4B (GE Healthcare, UK) in 1 mL of an organic solvent of 1-butanol/ethanol/H_2_O (4:1:1, *v*/*v*) and incubated for 45 min. The gel was washed twice with the organic solvent and incubated with an aqueous solvent of ethanol/H_2_O (1:1, *v*/*v*) for 30 min. The solution phase was recovered and dried using a SpeedVac concentrator. Glycopeptides were dissolved in 0.1% trifluoroacetic acid and desalted using a ZipTip C18 pipette tip (Millipore, Billerica, MA, USA).

#### 2.3.2. ESI-MS

The glycan profile of transferrin was obtained by liquid chromatography coupled with electrospray ionization (ESI) mass spectrometry (MS). The eluate from the immune-affinity column described above was directly injected into a small reversed phase column (Inertsil C4, GL Science, Tokyo, Japan) (0.2 × 3 mm). After washing the column with 0.1% (*v*/*v*) formic acid, the solvent was switched to 60% acetonitrile/0.1% formic acid and directly introduced into Q-STAR ESI time-of-flight (TOF)-MS (AB Sciex, Framingham, MA, USA). The multi-charged ion mass spectrum was transformed/deconvoluted into a singly-charged species by the ProMass deconvolution program (Novatia LLC, Newtown, PA, USA).

#### 2.3.3. MALDI-TOF-MS

A site-specific analysis of glycans was performed by matrix-assisted laser desorption/ionization (MALDI)-TOF-MS [19]. Enriched glycopeptides were dissolved in a 10 mg/mL 2,5-dihydroxybenzoic acid solution in 50% (*v*/*v*) acetonitrile and loaded onto the MALDI sample target plate of Voyager DE-Pro MS (AB Sciex). Measurements were performed in the positive ion and linear TOF mode.

### 2.4. WES and Sanger Sequencing

WES was performed on the children (II-1,2,3) and their parents (I-1, 2) using a previously described method [20]. Genomic DNA was obtained from peripheral blood leukocytes by standard methods, captured with the SureSelect Human All Exon Kit V5 (Agilent Technologies, Santa Clara, CA, USA), and sequenced on a HiSeq 1000 platform (Illumina, San Diego, CA, USA) with 101-bp paired-end reads. An image analysis and base calling were performed with a sequence control software real-time analysis and CASAVA software v1.8 (Illumina). Reads were aligned to the human genome assembly hg19 (GRCh37) as a reference with the Burrows–Wheeler Alignment tool 0.7.10. (http://bio-bwa.sourceforge.net/, accessed on 2 November 2021). Alignments were converted from a sequence alignment map (SAM) format to sorted and indexed binary alignment map files (SAMtools version 0.1.19). Duplicate reads were removed using Picard 1.118 (http://broadinstitute.github.io/picard/, accessed on 2 November 2021). Local realignments around insertions or deletions (indels) and base quality score recalibration were performed with the Genome Analysis Toolkit (GATK) 3.2–2 (https://gatk.broadinstitute.org/hc/en-us/, accessed on 2 November 2021). Single-nucleotide variants and small indels were identified using the GATK Unified Genotyper and filtered according to the Broad Institute’s best-practice guidelines. Variants that passed the filters were annotated using ANNOVAR (https://annovar.openbioinformatics.org/en/latest/, accessed on 2 November 2021). After merging the VCF files of all members of the family, we filtered variants with MAF > 0.05 from the 1000 Genomes Project databases (https://www.internationalgenome.org/, accessed on 2 November 2021), NHLBI GO Exome Sequencing Project (ESP6500, http://evs.gs.washington.edu/EVS/, accessed on 2 November 2021)), and the Human Genetic Variation Database (http://www.genome.med.kyoto-u.ac.jp/SnpDB/, accessed on 2 November 2021). Sanger sequencing around the identified variant was performed in both directions in the affected individuals and unaffected parents and sibling.

### 2.5. HPLC Analysis of Sialylated N-Linked Glycans Released from Total Plasma Proteins

In the analysis, 25-µL aliquots of plasma from healthy individuals (Controls 4, 5, and 6) and the patients (II-1 and 2) and their unaffected sibling (II-3) were mixed with 10 µL of 1 M ammonium bicarbonate, 1 µL of 1 M DTT, and 64 µL of Milli-Q water and then heated at 60 °C for 30 min. After the addition of 10 µL of 220 mM iodoacetamide dissolved in water, the mixture was allowed to stand at room temperature for 1 h. Twenty units of trypsin (Thermo) was added and incubated at 37 °C for 4 h, followed by heat inactivation at 100 °C for 5 min. Samples were subsequently treated with PNGase F (2 units, Roche, Basel, Switzerland) at 37 °C for 16 h followed by heat denaturation at 100 °C for 5 min. Three volumes of cold ethanol were added to the reaction mixtures, followed by centrifugation at 17,000× *g* at 4 °C for 15 min to remove proteins, and the supernatant was then dried in vacuo. Released *N*-glycans were desalted using a PD-10 column (GE Healthcare) and subjected to 2-aminopyridylation (PA-labeling) as previously described [21]. The structures of the standard Man8GlcNAc2 isomers A-C, obtained from Takara Bio Inc. (Shiga, Japan), are shown in Supplemental Figure S1. Note that the nomenclature of isomers A and B was swapped from the original name by the company, according to the common nomenclature.

PA-labeled glycans were analyzed by anion exchange chromatography using a TSKgel DEAE-5PW column (φ7.5 × 75 mm; Tosoh, Tokyo, Japan) to quantify the sialylated glycans as previously described [22]. The structures of standard Sia1-4, obtained from Takara Bio Inc., are shown in Supplemental Figure S2.

### 2.6. Subcellular Fractionation of Lymphocytes

Lymphocytes were collected from 10 mL of blood using Lymphosepar I (IBL, Fujioka, Japan) according to the manufacturer’s protocol. Cell disruption and subsequent fractionation steps were performed at 4 °C. Cells were resuspended in hypotonic buffer (10 mM HEPES-NaOH (pH 7.4), 10 mM KCl, and 5 mM MgCl_2_) and incubated for 10 min. Swollen cells were centrifuged and resuspended in 100 µL of the same buffer containing Complete protease inhibitor EDTA-free (Roche), and disrupted by three cycles of the freeze-thaw method followed by 20 passages through a 27-gauge needle. Homogenates were immediately mixed with 100 µL of 0.5 M sucrose in 10 mM HEPES-NaOH (pH 7.4), and nuclei and unbroken cells were then pelleted by centrifugation at 1000× *g* for 10 min. The postnuclear supernatant was centrifuged at 8000× *g* for 20 min, and the supernatant obtained was further centrifuged at 100,000× *g* for 90 min. The resulting pellet (microsome fraction) was used for biochemical assays.

### 2.7. Enzyme Assay

Microsome fractions were resuspended in 20 µL of reaction buffer (50 mM MES-NaOH (pH 6), 0.1% Triton X-100, 1 mM CaCl_2_, 10 μM swainsonine, and 2 pmol of PA-labeled Man_9_GlcNAc_2_ as the substrate), and incubated at 37 °C for 24 h. The reaction was terminated by heating at 100 °C for 5 min, and proteins were removed by ethanol precipitation. Size fractionation HPLC was performed using a Shodex NH2P-50 4E column (φ4.6 × 250 mm; Shodex, Tokyo, Japan) as previously described [21].

## 3. Results

### 3.1. MS

**Figure 2:**
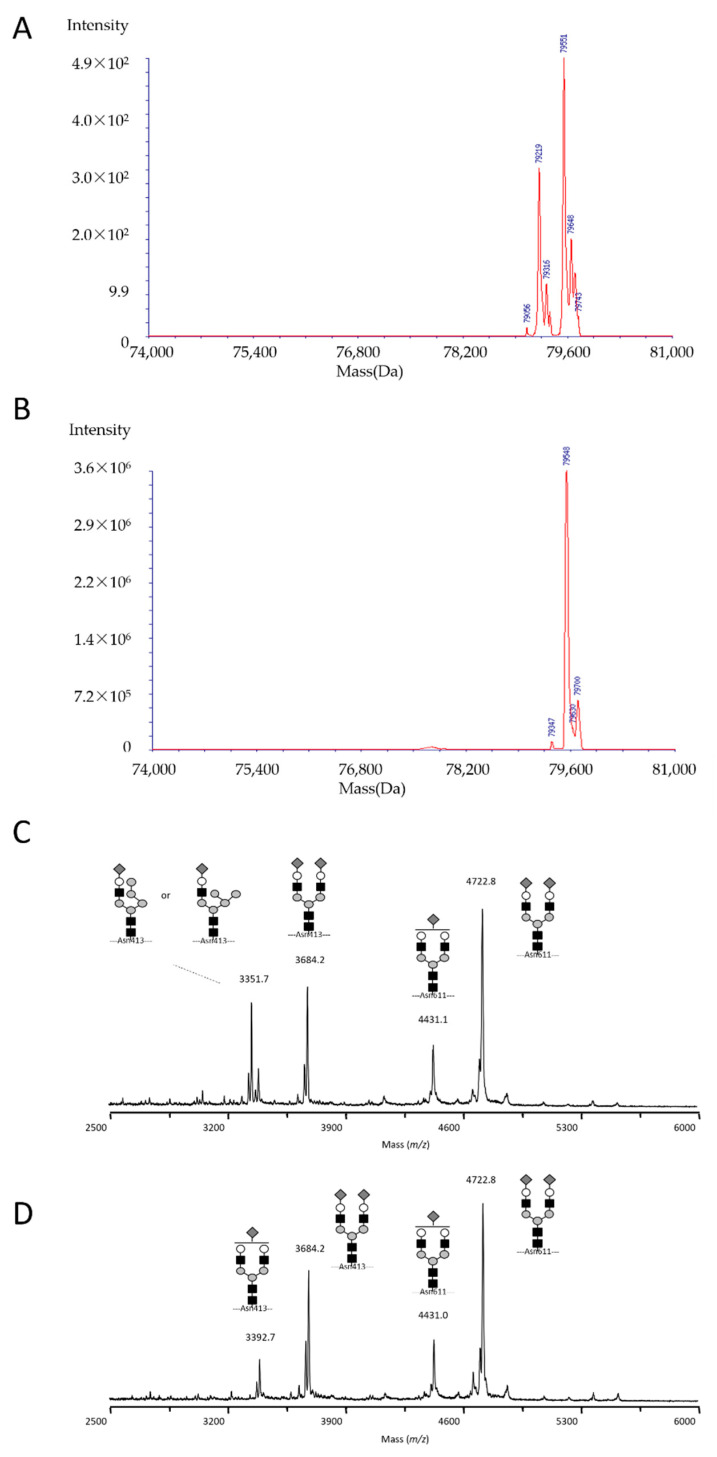
Mass spectrometry. Deconvoluted ESI mass spectra of transferrin from (**A**) Patient II-1 and (**B**) a healthy control, suggested the presence of a hybrid-type oligosaccharide on transferrin in the patient. MALDI mass spectra of glycopeptides from (**C**) the patient and (**D**) a healthy control indicated that the abnormal oligosaccharide was exclusively found at the Asn413, but not Asn611 glycosylation site. Similar results were obtained for patient II-2 (data not shown).

**Figure 3:**
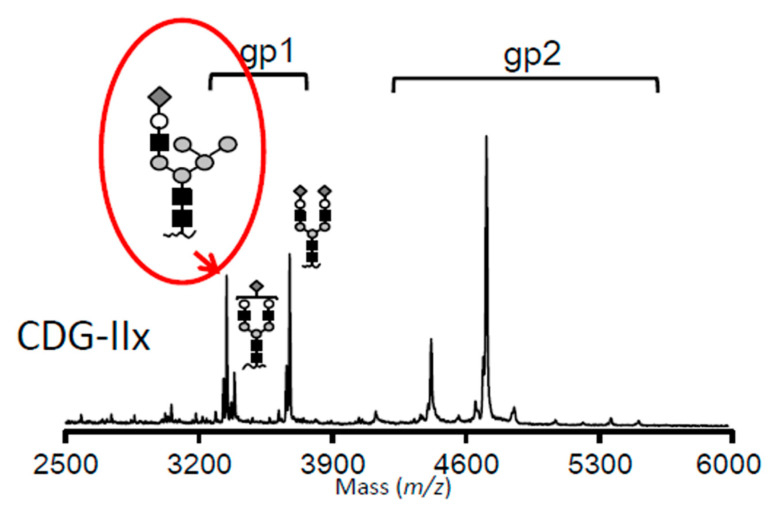
Mass spectrometry. The hybrid oligosaccharide containing a linear mannose strand was the more likely candidate in the structures given on the signature peak (see the text for an explanation).

We analyzed the structural features of glycans on serum transferrin using ESI-MS and MALDI-TOF. A deficiency in the carbohydrate chain was confirmed by MS. Patients had transferrin molecules with four, three, two, one, or no sialic acid residues, which is typical of CDG-type II defects (Figure 2A–D and Figure 3).

The molecular mass of serum transferrin was also analyzed by ESI-MS. Patient samples showed an isoform with a molecular mass of 79,219 and normal tetrasialylated transferrin with a molecular mass of 79,551 in the deconvoluted spectrum (Figure 2A), while the latter was the only major peak in the blood sample obtained from a healthy individual (Figure 2B). Mass differences between wild and abnormal molecules calculated from original multi-charged mass spectra were 331.0 ± 1.2 and 331.9 ± 1.5 Da (mean ± SD, *n* = 10) for patients II-1 and II-2, respectively (see Supplemental Figure S1). This difference may be attributed to alterations in the glycan moiety rather than a polymorphism in the protein backbone sequence. Furthermore, the decrease of 330–333 Da suggested the replacement of the hybrid for a biantennary oligosaccharide. The multi-charged mass spectra of serum transferrin from patients II-1 and II-2 are shown in Supplemental Figure S3.

Since the mass of the abnormal isoform suggested the presence of a hybrid-type oligosaccharide at one of the two glycosylation sites of transferrin (i.e., Asn413 and Asn611), a site-specific analysis was conducted by MALDI-TOF-MS. Interestingly, the hybrid-type glycan was detected at Asn413, but not at Asn611 (Figure 2C). This may have been due to a difference in the accessibility of the MAN1B1 enzyme to the oligosaccharide in the nascent glycoprotein molecule. Therefore, the glycan on Asn611 may be more accessible to glycan-processing enzymes in the Golgi. In Figure 3, two candidate structures were available for the hybrid-type oligosaccharide. Since MAN1B1 removes the terminally α1,2-linked mannose in the middle arm of the substrate Man_9_GlcNAc_2_ oligosaccharide [23,24], the structure with a linear mannose was more plausible.

### 3.2. WES and Sanger Sequencing

**Figure 4:**
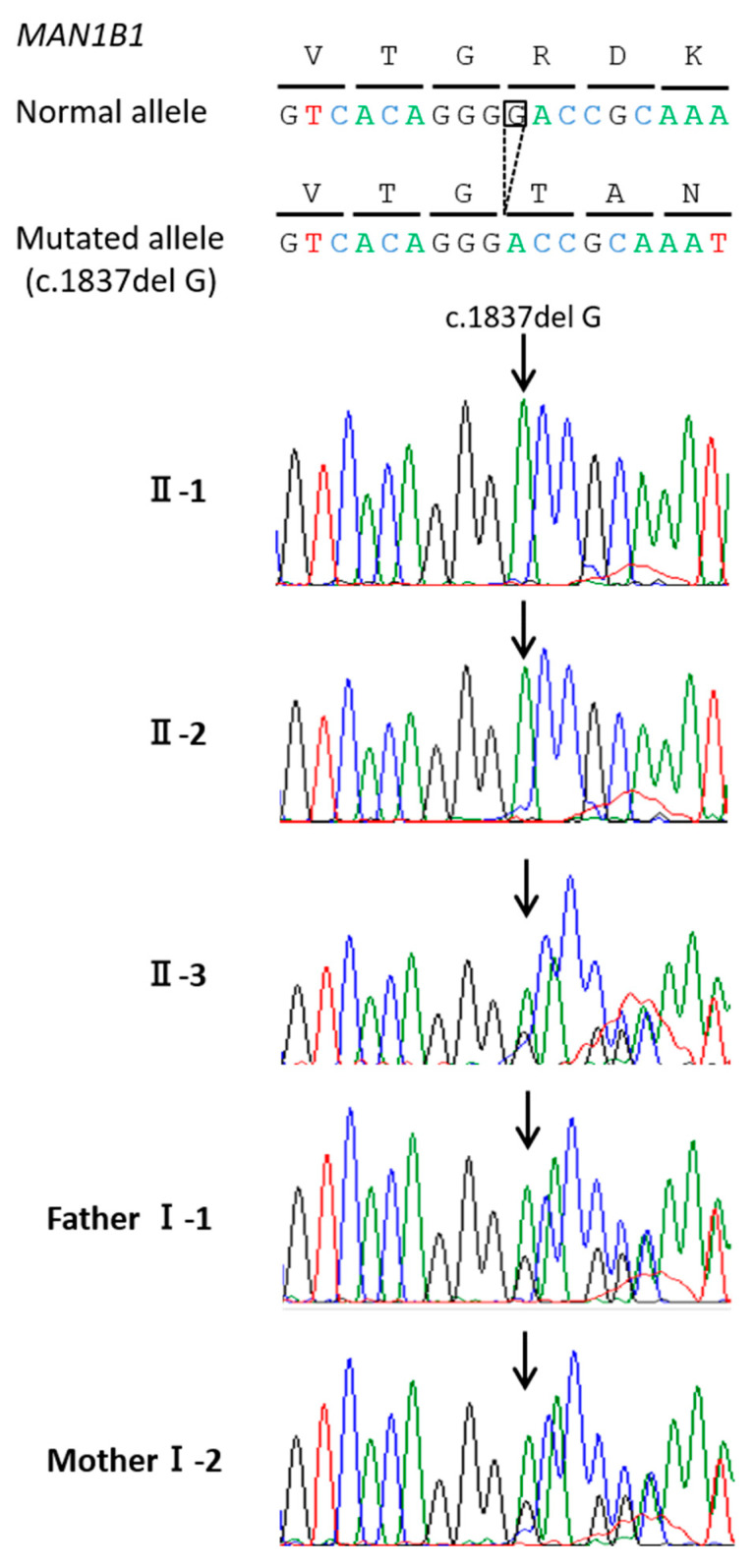
Sanger sequencing: Patients II-1 and II-2 were homozygous for the variant (*MAN1B1*: exon 12, c.1837del, p.Asp613Thrfs*115). Their parents and unaffected sister (II-3) were heterozygous carriers.

WES was performed to identify the gene causing the CDG-II deficiency. After the identification of variants and filtering out variants present in the various public population databases and synonymous SNPs, we focused on non-synonymous variants, splice acceptor and donor site variants, and short, frameshift coding indels, which are more likely to be pathogenic variants. Only one variant, NM_016219.4(MAN1B1_v001):c.1837del in exon 12 of *MAN1B1*, which caused a frameshift at codon 613, resulting in a longer protein through the loss of the original stop codon (NM_016219.4(MAN1B1_i001):p.(Asp613Thrfs*115)), was a homozygous variant. This resulted in the loss of part of the region of Glycosyl hydrolase family 47 (pfam01532) associated with glycosyl hydrolase activity, which is expected to lead to the loss of protein function; therefore, we concluded that this variant was the disease-causing alteration in our patient (Mutation Taster, http://www.mutationtaster.org/ accessed on 2 November 2021). Sanger sequencing confirmed that this frame-shift mutation was homozygous in the two affected patients (II-1 and II-2), and heterozygous in both parents (I-1 and I-2) and the unaffected sibling (II-3) (Figure 4). This variant has not been reported in any patients with CDG (Human Gene Mutation Database professional, http://www.hgmd.org/ accessed on 2 November 2021, and ClinVar, http://www.ncbi.nlm.nih.gov/clinvar/ accessed on 02 November 2021). The American College of Medical Genetics and Genomics guidelines [25] classified this variant as pathogenic (PVS1, PM2, and PP4).

### 3.3. Sialylated N-Linked Glycan Profile of Total Plasma Proteins

**Figure 5:**
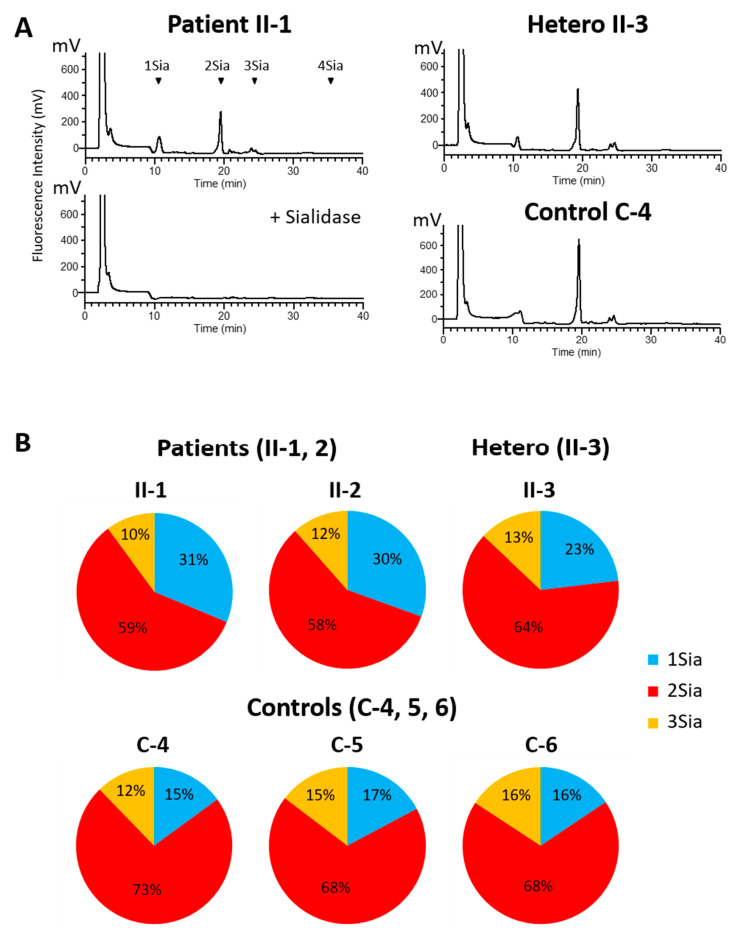
Sialylated N-linked glycan profile of total plasma proteins. (**A**,**B**) PA-derivatized N-glycans prepared from pedigree (II-1, 2, 3) or control (C-4, 5, 6) plasma were analyzed by anion exchange HPLC to elucidate sialylation profiles. In MAN1B1-deficient patients (II-1, 2), we observed the accumulation of monosialoglycans, indicating N-glycan-processing defects. Controls showed a normal pattern. The heterozygous carrier, the healthy sister (II-3), showed an intermediate level.

PA-derivatized N-glycans prepared from pedigree (II-1, 2, 3) or control (C-4, 5, 6) plasma were analyzed by anion exchange HPLC to elucidate sialylation profiles. Monosialoglycan levels were higher in MAN1B1-deficient patients (II-1, 2) than in the controls, strongly indicating N-glycan-processing defects (Figure 5A,B). The unaffected sibling (II-3), who carries a heterozygous mutation, showed an intermediate phenotype between the patients (II-1, 2) and healthy controls (C-4, 5, 6), indicating that the MAN1B1 reaction is one of the rate-limiting points for the formation of complex-type N-glycans.

### 3.4. Characterization of the 1,2-Mannosidase I Activity of the Lymphocyte Microsome Fraction

**Figure 6:**
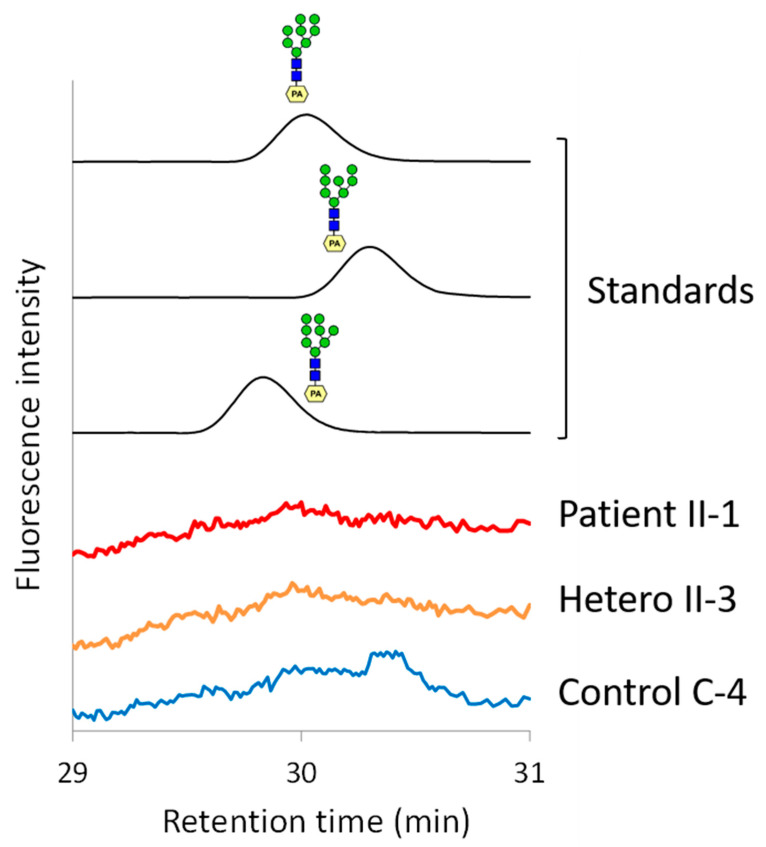
Elution profile of standard Man8GlcNAc2–PA isomers A-C and representative profiles for the product of the alpha1,2-mannosidase activity assay using the lymphocyte microsome fraction. The microsome fraction of control individuals generated Man8GlcNAc2–PA isomers B. In contrast, consistent with the MAN1B1 deficiency, the formation of Man8GlcNAc2–PA isomer B was absent in the microsome fraction of lymphocytes from the patients (II-1, 2). All profiles from the patients (II-1,2), unaffected sibling (II-3), and control samples (C-4–6) are shown in Supplemental Figure S4.

MAN1B1 and other class 1 α1,2-mannosidases are members of glycosyl hydrolase family 47, and are considered to be key enzymes for the structural remodeling of *N*-linked glycans on proteins that pass through the secretory pathway. MAN1B1 specifically catalyzes the trimming of the B-arm (middle branch) mannose residue of Man_9_GlcNAc_2_ to generate Man_8_GlcNAc_2_ isomer B, while MAN1A1 (also called Golgi α1,2-mannosidase IA) predominantly forms Man_8_GlcNAc_2_ isomer A [23,24]. The mannosidase activities present in the microsome membranes prepared from lymphocytes were examined by an incubation with Man_9_GlcNAc_2_–PA as a substrate. While only negligible α1,2-mannosidase activities were detected in the microsome fractions of lymphocytes, samples from control individuals, particularly samples from C-4 and C-6, yielded two distinct peaks, the latter being that of the B isoform formed by MAN1B1 (Figure 6, Supplemental Figure S4). In contrast, consistent with the MAN1B1 deficiency, the formation of Man_8_GlcNAc_2_–PA isomer B was not detected in either patient (II-1 and II-2). This result further supported the activity of MAN1B1 being compromised in patient-derived lymphocytes.

## 4. Discussion

We herein report siblings with MAN1B1-CDG. CDG was diagnosed by MS and WES. The mass pattern from patients showed CDG-type II defects. WES revealed a novel pathogenic variant in *MAN1B1*.

The 15-year-old brother showed dysmorphic features, hypotonia, ID, West syndrome, and the decreased activity of blood coagulation factors. The 11-year-old sister developed cerebral infarction in MCA during the neonatal period. Dysmorphic features, hypotonia, and psychomotor retardation were also noted. Neither sibling had truncal obesity, a unique finding in MAN1B1-CDG. They also showed a very mild elevation in AST and decreased protein C and protein S activities during infancy.

Rafiq et al. [3] reported variants of *MAN1B1* in patients with non-syndromic autosomal-recessive intellectual disability (NS-ARID). Four of the families were from Pakistan, and one was from Iran. One Pakistani family with ID and dysmorphic features had a homozygous nonsense variant (p.Trp473*). The other three families had a missense variant, p.Glu397Lys, which segregates with NS-ARID. The three families come from the same village and may have shared inheritance. In the Iranian family, the missense variant p.Arg334Cys also segregated with NS-ARID. However, abnormalities in the glycosylation pattern were not described.

Rymen et al. [5] reported seven patients with similar clinical features (developmental delay, ID, facial dysmorphism, and obesity) due to a MAN1B1 deficiency. Dysmorphic features, including down-slanting palpebral fissures, hypertelorism, large low-set ears, a hypoplastic nasolabial fold, and a thin upper lip, were noted. Their findings implicated a MAN1B1 deficiency in the development of N-glycosylation disorders and CDG type II syndrome. The MAN1B1 protein was originally considered to reside in the ER, and, thus, is called ER α-mannosidase I [23,24]. However, Rymen et al. [5] confirmed that the MAN1B1 protein localized to the Golgi apparatus. They suggested that MAN1B1 controls protein quality at the level of the Golgi apparatus. Alterations in the Golgi structure were detected in these patients.

Van Scherpenzeel et al. [6] revealed a pathogenic variant of *MAN1B1* in a patient with ID by WES. A novel MS method was applied for the high-resolution glycoprofiling of transferrin. A highly characteristic glycosylation signature was observed with hybrid-type N-glycans. They subsequently screened 100 patients with CDG type II, identified 11 additional patients with the MAN1B1-CDG profile, and detected *MAN1B1* pathogenic variants. Saldova et al. [8] identified and quantified novel hybrid high-mannosylated MAN1B1-CDG-specific IgG glycans and found an increase in sialyl Lewis x (sLex) glycans in the serum proteins of MAN1B1-CDG patients. Balasubramanian et al. [12] reported two families with MAN1B1-CDG though WES. These families, each with two siblings, showed a variable level of ID. They also exhibited characteristic facial dysmorphism, hypotonia, truncal obesity and, in some, behavioral issues.

Kasapkara et al. [16] reported three patients with MAN1B1-CDG, all of whom had presented due to dysmorphic and neurological findings, and prominent hypertransaminasemia was detected. Van Scherpenzeel et al. [6] detected mild elevations in liver function tests in two out of eight patients. Although the cause of hypertransaminasemia was not clear, there was no evidence of an infection or underlying liver pathology. Our patients also showed hypertransaminasemia. Therefore, hypertransaminasemia appears to be one of the important findings in MAN1B1-CDG patients. A transient elevation in the serum level of ALP is a novel result.

In comparisons with PMM2-CDG [26], systemic features are not common in MAN1B1-CDG patients. However, our patients showed unique manifestations of MAN1B1-CDG. Patient II-1 developed West syndrome when he was 8 months old, which was successfully treated. Seizures have been reported in several cases of MAN1B1-CDG. Early-onset epileptic encephalopathy may be a novel complication. Patient II-2 presented with MCA infarction in the neonatal period. SLE are a potential complication of PMM2-CDG, and have been reported in at least 36 patients [26]. SLE in MAN1B1-CDG were previously described by Kemme et al. [14]. Izquierdo-Serra et al. [27] proposed that the hypoglycosylation of CaV2.1 encoded by CACNA1A as a novel pathomechanism of SLE and ataxia in PMM2-CDG. A similar mechanism may also be involved in MAN1B1-CDG. In addition, coagulation abnormalities may have contributed to MCA infarction in patient II-2. Coagulation abnormalities, including decreased protein C and protein S activities, were noted in the siblings. Mild coagulation abnormalities were previously reported in MAN1B1-CDG [8].

Collectively, the present results indicate that the clinical manifestations of MAN1B1-CDG show more variability. Epileptic seizures, including West syndrome, and coagulopathy resulting in strokes may be important complications of MAN1B1-CDG. An analysis of the glycosylation pattern of serum transferrin is important in patients with unknown neurological abnormalities.

## 5. Conclusions

The present patients with MAN1B1-CDG showed unique manifestations, including early-onset epileptic encephalopathy and MCA infarction. Truncal obesity, which is one of the characteristic features of MAN1B1-CDG, was not observed. We conducted a HPLC analysis of sialylated N-linked glycans released from total plasma proteins and characterized the α1,2-mannosidase I activity of the lymphocyte microsome fraction. We detected the accumulation of monosialoglycans, indicating N-glycan-processing defects. The enzymatic activity of MAN1B1 was compromised in patient-derived lymphocytes. The present study expands the clinical spectrum of MAN1B1-CDG and addresses the methods required to investigate its pathogenesis.

## Data Availability

Not applicable.

## Supplementary Materials

The following are available online at https://www.mdpi.com/article/10.3390/cells10113117/s1, Figure S1: Structures of standard PA-labeled glycans used in this study. No. indicates the product number of Takara Bio Inc. Green circle, yellow circle, blue square and purple diamond indicates mannose, galactose, GlcNAc and NeuAc, respectively. Anomery of the linkages as well as the linkage of core structures (Manb1,4-GlcNAcb1-4GlcNAc) have been omitted. Figure S2: Structures of standard Sia1-4, obtained from Takara Bio Inc., are shown., Figure S3: Multiply-charged mass spectra of serum transferrin from patients II-1 (upper) and II-2 (lower). The mass difference between wild and abnormal transferrin molecules was calculated from each of 31 to 39-charged species, and it was 331.0 ± 1.2 Da and 331.9 ± 1.5 Da (mean ± SD, *n* = 10), for patient II-1 and II-2, respectively. Figure S4: HPLC profiles of the 1,2-mannosidase I activity assay using the lymphocyte microsome fraction. II-1,2; microsome fraction of lymphocytes from the patients; II-3 microsome fraction of lymphocytes from the unaffected sibling; Control 4–6; microsome fraction of lymphocytes from healthy controls (C-4–6).
