# Supplementary material for: Siblings with MAN1B1-CDG Showing Novel Biochemical Profiles
Source: Cells. 2021 Nov 10;10(11):3117. doi: 10.3390/cells10113117 (PMC8618856; doi:10.3390/cells10113117)
Supplement: Supplementary file 1 [file cells-10-03117-s001.zip › cells-1420688-supplementary.pptx]

## Slide 1
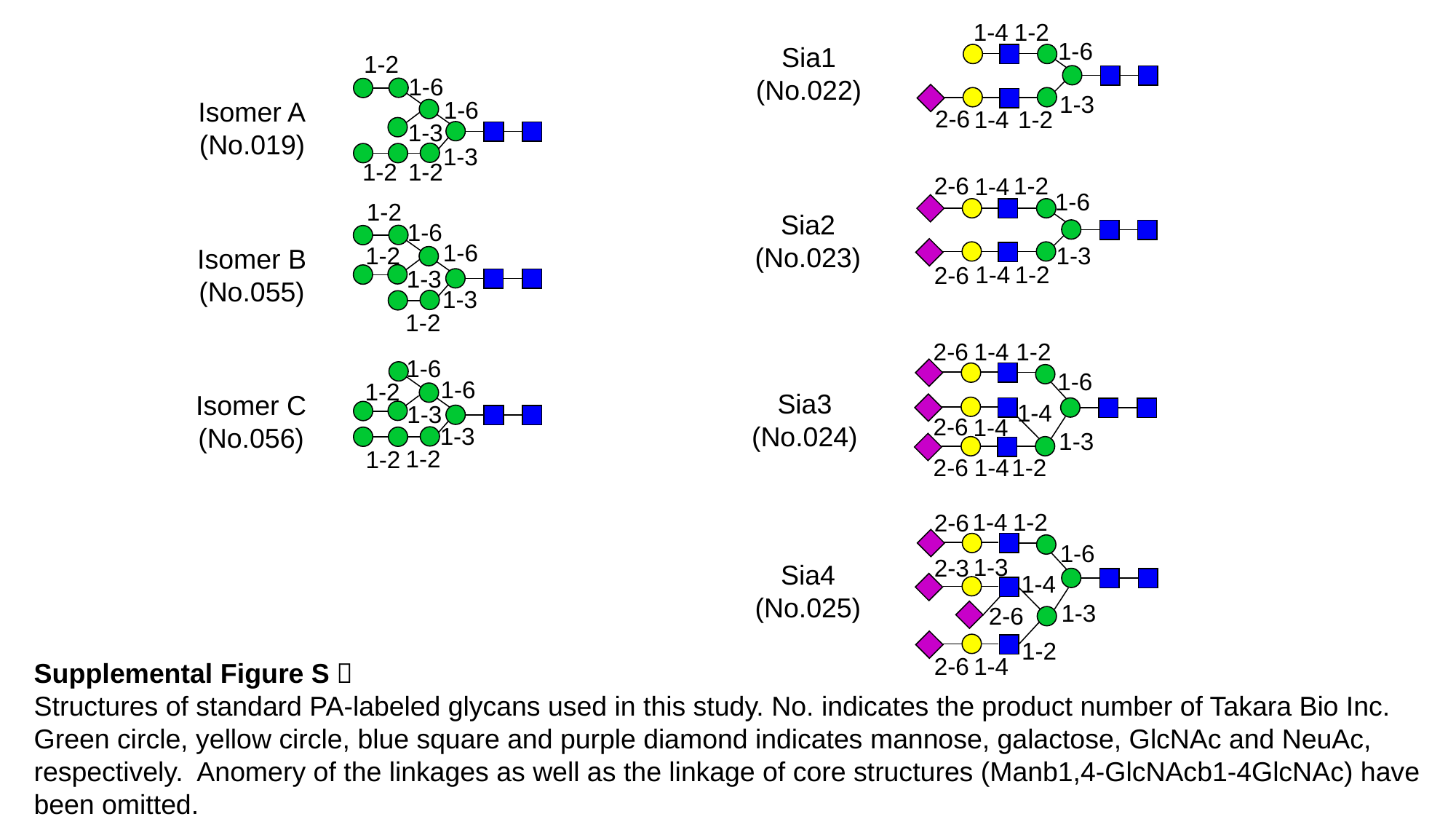

1-2
1-4
1-6
Sia1
(No.022)
1-2
1-6
1-3
Isomer A
(No.019)
1-6
2-6
1-4
1-2
1-3
1-3
1-2
1-2
1-2
2-6
1-4
1-6
1-2
Sia2
(No.023)
1-6
1-6
1-2
1-3
Isomer B
(No.055)
1-4
1-2
2-6
1-3
1-3
1-2
1-2
1-4
2-6
1-6
1-6
1-6
1-2
Sia3
(No.024)
Isomer C
(No.056)
1-4
1-3
2-6
1-4
1-3
1-3
1-2
1-2
1-4
1-2
2-6
1-2
1-4
2-6
1-6
1-3
2-3
Sia4
(No.025)
1-4
1-3
2-6
1-2
2-6
1-4
Supplemental Figure S１
Structures of standard PA-labeled glycans used in this study. No. indicates the product number of Takara Bio Inc. Green circle, yellow circle, blue square and purple diamond indicates mannose, galactose, GlcNAc and NeuAc, respectively. Anomery of the linkages as well as the linkage of core structures (Manb1,4-GlcNAcb1-4GlcNAc) have been omitted.

## Slide 2
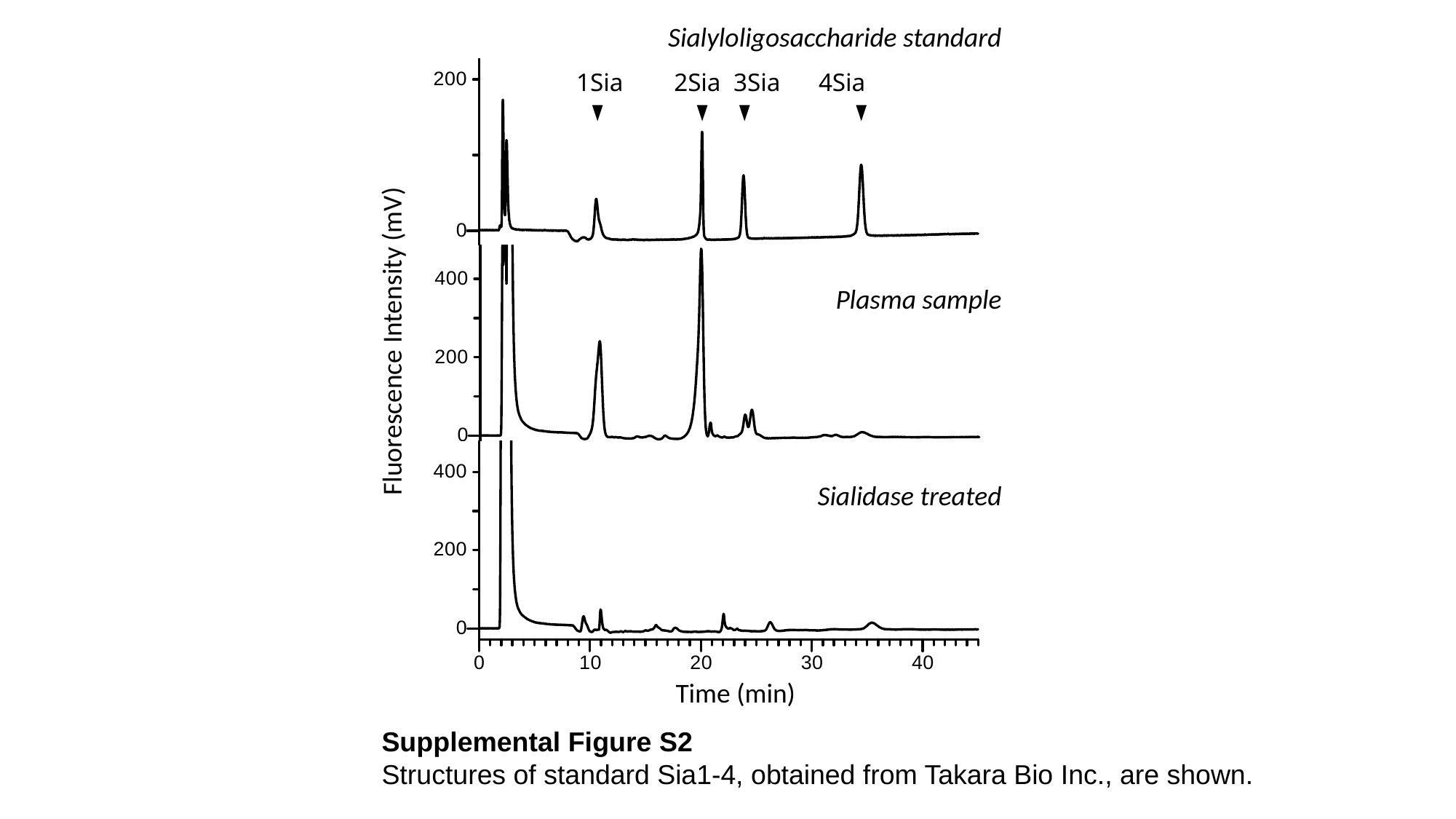

Sialyloligosaccharide standard
Plasma sample
Sialidase treated
1Sia 2Sia 3Sia 4Sia
Fluorescence Intensity (mV)
Time (min)
Supplemental Figure S2
Structures of standard Sia1-4, obtained from Takara Bio Inc., are shown.

## Slide 3
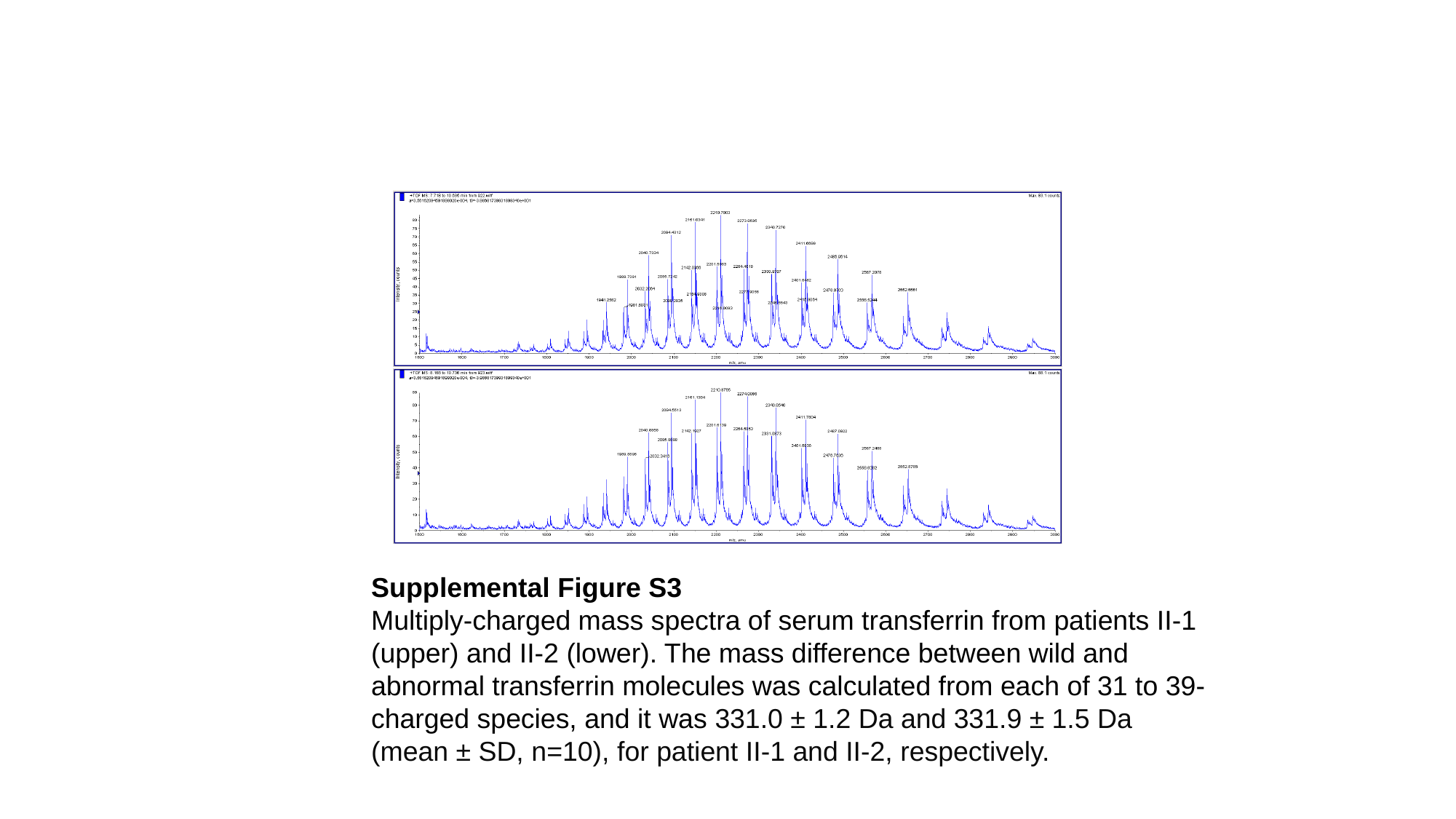

Supplemental Figure S3
Multiply-charged mass spectra of serum transferrin from patients II-1 (upper) and II-2 (lower). The mass difference between wild and abnormal transferrin molecules was calculated from each of 31 to 39-charged species, and it was 331.0 ± 1.2 Da and 331.9 ± 1.5 Da (mean ± SD, n=10), for patient II-1 and II-2, respectively.

## Slide 4
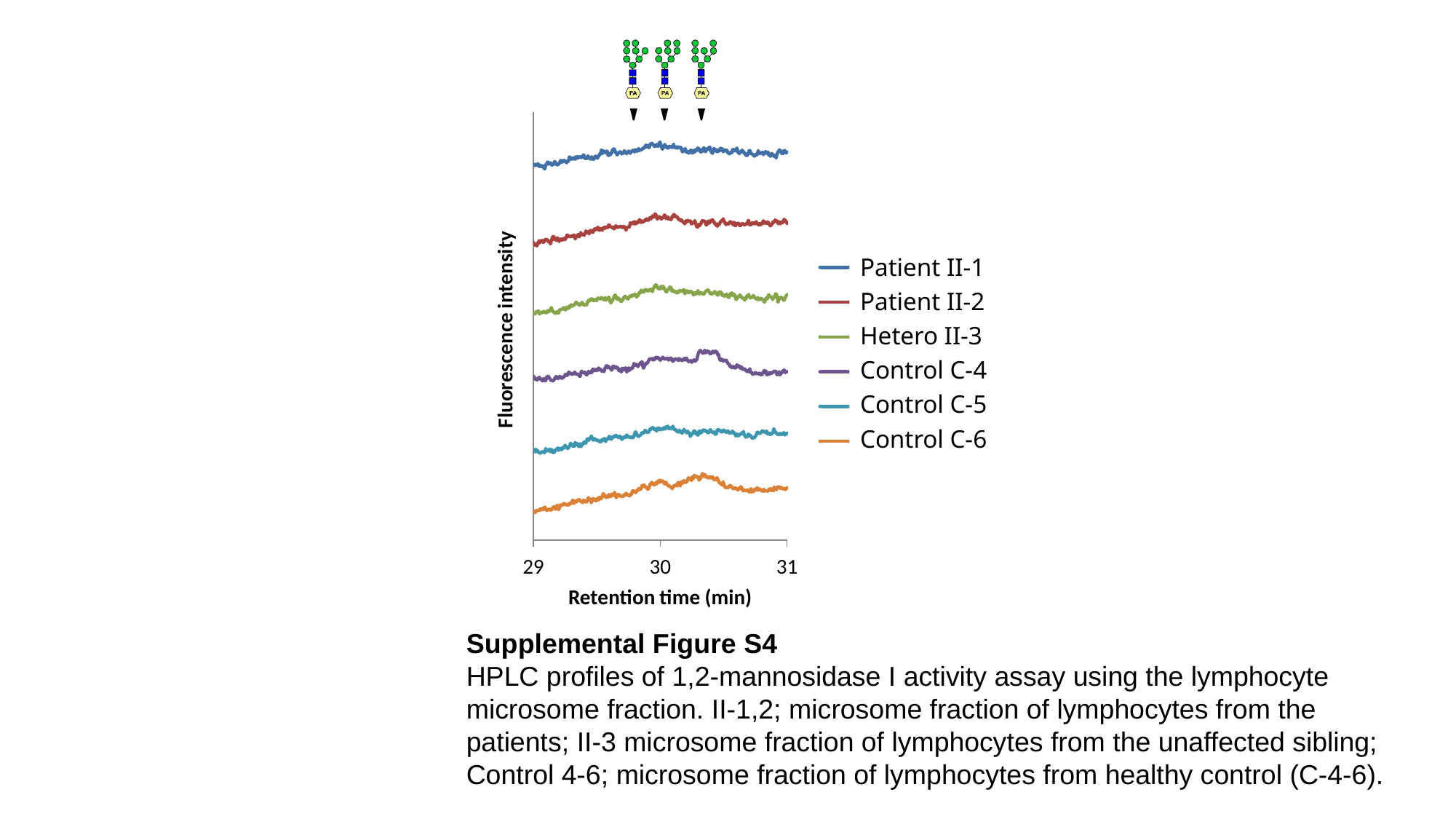

Patient II-1
Patient II-2
Hetero II-3
Control C-4
Control C-5
Control C-6
Supplemental Figure S4
HPLC profiles of 1,2-mannosidase I activity assay using the lymphocyte microsome fraction. II-1,2; microsome fraction of lymphocytes from the patients; II-3 microsome fraction of lymphocytes from the unaffected sibling; Control 4-6; microsome fraction of lymphocytes from healthy control (C-4-6).
